# Wound Botulism Outbreak Among Persons Who Use Black Tar Heroin — San Diego County, California, 2017–2018

**DOI:** 10.15585/mmwr.mm675152a3

**Published:** 2019-01-04

**Authors:** Corey M. Peak, Hilary Rosen, Amanda Kamali, Alyssa Poe, Mahtab Shahkarami, Akiko C. Kimura, Seema Jain, Eric McDonald

**Affiliations:** ^1^Epidemic Intelligence Service, CDC; ^2^County of San Diego Health and Human Services Agency, California; ^3^Division of Global Migration and Quarantine, National Center for Emerging and Zoonotic Infectious Diseases, CDC; ^4^Infectious Diseases Branch, California Department of Public Health; ^5^Microbial Diseases Laboratory, California Department of Public Health.

During September 29–October 6, 2017, the County of San Diego Public Health Services (COSD) was notified of two patients with suspected wound botulism and a history of using black tar heroin. On October 9, COSD, which had reported an average of one wound botulism case per year during 2001–2016, sent a health alert through the California Health Alert Network, notifying Southern California providers of these two patients, including their signs and symptoms and black tar heroin exposure. In collaboration with the California Department of Public Health, COSD conducted an investigation to identify additional cases, determine risk factors for illness, estimate cost of medical care, and develop recommendations to prevent further illness. By April 18, 2018, nine (eight confirmed and one probable) patients with wound botulism were identified, all of whom were hospitalized; one of the nine died. All nine were persons who inject drugs; seven specifically reported using black tar heroin and six practiced subcutaneous injection known as skin popping. Clinically compatible signs and symptoms included muscle weakness, difficulty swallowing, blurred vision, drooping eyelids, slurred speech, difficulty breathing, loss of facial expression, or descending paralysis. All patients were treated with heptavalent botulism antitoxin (BAT). Wound botulism is likely underrecognized because of its rarity and the overlapping signs and symptoms with opioid intoxication, overdose, and other neurologic syndromes including Guillain-Barré syndrome, the Miller Fisher variant of Guillain-Barré syndrome, and myasthenia gravis. Prompt diagnosis, administration of BAT, and provision of supportive care can help stop the progression of paralysis and be lifesaving.

## Investigation and Results

A confirmed case was defined as illness in a resident of San Diego County who had 1) clinically compatible signs or symptoms of botulism during September 2017–May 2018; 2) laboratory detection of botulinum neurotoxin (BoNT) in serum; 3) a history of injection drug use during the 2 weeks before illness onset; and 4) no suspected exposure to a contaminated food. A probable case was defined similarly, but without laboratory confirmation. All wound botulism patients reported to COSD were asked by investigators about potential exposures using a standardized questionnaire. Self-reported history of injection drug use was recorded for each patient, with drug use corroborated by toxicology results when possible. Serum collected from each patient was tested for BoNT by mouse bioassay at the California Department of Public Health’s Microbial Diseases Laboratory; serum specimens with indeterminate results were tested by matrix-assisted laser desorption/ionization time-of-flight mass spectrometry at CDC. Direct hospital charges for the outbreak-associated patients were estimated based on hospital charges for wound botulism cases reported to COSD during 2005–2016 from the California Office of Statewide Health Planning and Development database.*

Among nine total cases, eight patients were men; median age was 40 years (range = 25–67 years). Symptom onset dates ranged from September 26, 2017, (epidemiologic week 39) to April 12, 2018 (epidemiologic week 15) (Figure). The most frequently reported symptoms were muscle weakness, difficulty swallowing, and blurred vision (Table 1). Abscesses were observed for five patients. Symptoms of wound botulism were initially attributed to drug intoxication for four patients. One patient was admitted for 7 days before receiving BAT and died 9 days later at a long-term care facility. One patient had received the opioid overdose reversal medication naloxone without improvement in symptoms, and one patient had received 2 doses of naloxone upon admission after at least one previous emergency department visit associated with wound botulism. A fourth patient, who was evaluated for symptoms of wound botulism and a history of close contact with a person known to have wound botulism, was discharged from the hospital before later being readmitted. All nine patients required admission to the intensive care unit; six required endotracheal intubation and mechanical ventilation, one of whom died. Median duration of hospitalization was 15 days (range = 9–67 days) until discharge to long-term care facilities (eight, including the patient who died) or departure against medical advice (one). All patients reported history of injecting heroin; seven reported using black tar heroin, six injected heroin by skin popping, and one patient did not report injection method. Toxicology tests performed for six patients were all positive for opioids. Two patients reported close contact with each other that included sharing drugs and needles.

**FIGURE:**
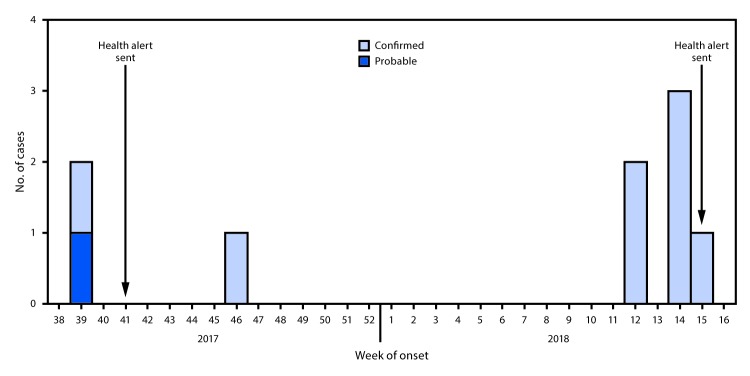
Confirmed and probable wound botulism cases, by epidemiologic week of symptom onset — San Diego County, California, September 2017–April 2018

**TABLE 1:** Characteristics of wound botulism cases (N = 9) — San Diego County, California, 2017–2018

Characteristic	No. (%) of patients
**Sign/Symptom**	
Subjective muscle weakness	9 (100)
Difficulty swallowing	8 (89)
Blurred vision	8 (89)
Drooping eyelids	7 (78)
Slurred speech	7 (78)
Difficulty breathing	5 (56)
Double vision	5 (56)
Descending paralysis	5 (56)
Abscess	5 (56)
**Complication**	
Hospitalization	9 (100)
Endotracheal intubation/Mechanical ventilation	6 (67)
Death	1 (11)
**Self-reported illicit drug use**	
Heroin	9 (100)
Intravenous injection	9 (100)
Black tar heroin	7 (78)
Subcutaneous injection (skin popping)	6 (67)

In coordination with COSD, the California Department of Public Health authorized BAT, which was released for nine patients by CDC quarantine stations in Los Angeles (eight) and San Francisco (one). Median interval from symptom onset to BAT administration was 6.5 days (range = 2.7–10.5 days) (Table 2). Pre-BAT serum specimens from nine patients were collected for testing; BoNT type A was confirmed for six patients by mouse bioassay and two patients by matrix-assisted laser desorption/ionization time-of-flight mass spectrometry. BoNT was not detected for one patient; however, that serum sample was frozen and hemolyzed and therefore not in optimal condition for confirmatory testing.

**TABLE 2:** Timing of events among patients with wound botulism (N = 9) — San Diego County, California, 2017–2018

Event timing	Median no. of days (range)
Illness onset to hospital admission	2.0 (0.1–6.0)
Hospital admission to BAT request	2.5 (0.1–9.1)
BAT request to BAT administration	0.2 (0.2–0.4)
Illness onset to BAT administration	6.5 (2.7–10.5)
Duration of hospitalization	15 (9.0–67.0)

**Abbreviation:** BAT = botulism antitoxin.

During the 2017–2018 outbreak, all nine patients were enrolled in public health care programs, including Medi-Cal^†^ (seven), Medicare (one), and the Veterans Health Administration (one). The total direct hospital costs for this outbreak was estimated at $2.3 million, for 203 total in-patient days charged at the historical median daily rate of $11,506 per day, based on data available for nine patients hospitalized with wound botulism in San Diego County during 2005–2016 (COSD, unpublished data; 2018).

## Public Health Response

Health alerts issued by COSD on October 9, 2017, and April 10, 2018, reminded health care providers to educate persons who inject drugs about the risks and symptoms of wound botulism, thoroughly search for wounds, consider a wound botulism diagnosis for patients with injection drug use history and cranial nerve abnormalities or descending paralysis, and consult promptly with local health departments to request BAT (*1*,*2*). Within 1 day of the April 2018 health alert, local clinicians reported suspected clinical wound botulism for two currently hospitalized patients. Additional public health communications included presentations to the local infectious diseases medical society, the local chapter of the American College of Surgeons, and the local anti-opioid misuse coalition, and distribution of informational flyers at substance abuse, needle exchange, and methadone clinics. The California Department of Public Health issued a communicable disease brief to local health departments throughout California.

## Discussion

Botulism, a nationally notifiable condition, is a rare but serious illness of descending paralysis most commonly caused by the neurotoxin produced by the anaerobic, gram-positive bacteria *Clostridium botulinum;* wound botulism in particular results from germination of *C. botulinum* spores in a wound or other necrotic tissue (*3*,*4*). The 2017–2018 outbreak of wound botulism among persons who inject drugs in San Diego County was associated with black tar heroin use, possibly through contamination of one or more batches. Black tar heroin use poses a heightened risk for wound botulism attributable to its production, preparation, and practice. Black tar heroin is a dark, gummy drug primarily produced in Mexico and often contains adulterants to increase bulk or contaminants introduced during illicit transport to the United States, such as inside car tires or other unsanitary locations where the drug might be exposed to soil containing *C. botulinum* spores (*3*). Preparation of black tar heroin for injection through cooking does not destroy *C. botulinum* spores, which can survive high heat and later germinate to produce BoNT (*5*). Skin popping can create an anaerobic environment of necrotic tissue in which BoNT can be readily formed and released (*6*).

With recent increases in opioid misuse nationwide (*7*) there is a growing need for awareness of the risks and symptoms of wound botulism among persons who inject drugs. During 2001–2016, in the United States, 353 wound botulism cases were reported to CDC (*8*); 291 (82%) were from California, including 15 from San Diego County. Although rarely reported outside California, wound botulism likely is underdiagnosed in the United States (*5*). Diagnosing wound botulism can be challenging because of the complex testing required and symptoms that can overlap with other neurologic syndromes or opioid intoxication and overdose (*5*,*6*). In addition, law enforcement authorities throughout the western United States and increasingly in the northeast have confiscated black tar heroin (*9*), providing evidence of potential exposure to this primary risk factor for wound botulism (*3*).

Prompt BAT administration can help stop progression of paralysis (*10*). The median interval between symptom onset and BAT administration in this outbreak (6.5 days) primarily comprised the time from symptom onset to hospital admission (2.0 days) and a suspicion of botulism that prompted a BAT request (2.5 days). Consistent with a previous report (*5*), costs of inpatient medical care were high and paid at public or hospital expense because the patients lacked private medical insurance. Efforts to improve botulism prevention, identification, and prompt treatment can improve morbidity and mortality outcomes as well as likely lower the monetary burden to the public and health care system (*5*).

Persons who have symptoms of wound botulism should promptly seek medical care and communicate their specific drug practices to aid diagnosis and accelerate BAT administration. Persons who inject drugs should be aware that, although safe injection practices can reduce the risk for some bloodborne infections (e.g., human immunodeficiency virus and hepatitis), wound botulism remains a risk when injecting or skin popping black tar heroin.^§^ Clinicians caring for persons who inject drugs or persons who fail to respond to naloxone need to perform thorough searches for wounds, be alert for wound botulism, and inform patients of this potentially lethal consequence of injection drug use. Health departments can deliver these health messages and emphasize the importance of opioid overdose education, referral of persons who inject drugs to medication-assisted treatment for opioid use disorder, and implement timely surveillance and notification of injection drug users when wound botulism clusters are detected.

**Summary**

**What is already known about this topic?**

Wound botulism is a rare but serious illness associated with black tar heroin use, especially by subcutaneous injection (skin popping).

**What is added by this report?**

During September 2017–April 2018, nine cases of wound botulism were reported in San Diego County, California; all patients reported injecting heroin, and seven used black tar heroin, including subcutaneous injection in six patients. Symptoms were first attributed to drug intoxication for four patients; two received the opioid overdose reversal medication naloxone without improvement in symptoms. One patient died.

**What are the implications for public health practice?**

Increasing use of black tar heroin during the opioid crisis might lead to additional cases of wound botulism. Heightened awareness of the disease might improve timely diagnosis and treatment. Prompt diagnosis and administration of botulism antitoxin can be lifesaving.

**Table:** 

## Footnotes

* Search included *International Classification of Diseases, Ninth Revision* codes 040.4, 040.41, 040.42, and 005.1 and *International Classification of Diseases, Tenth Revision* codes A48.5, A48.51, A48.52, and A05.1.

† A program that offers low-cost or free health coverage to eligible California residents with limited income.
